# The Molecular Basis of Inactivation of Metronidazole-Resistant *Helicobacter pylori* Using Polyethyleneimine Functionalized Zinc Oxide Nanoparticles

**DOI:** 10.1371/journal.pone.0070776

**Published:** 2013-08-08

**Authors:** Soumyananda Chakraborti, Saurabh Bhattacharya, Rukhsana Chowdhury, Pinak Chakrabarti

**Affiliations:** 1 Department of Biochemistry, Bose Institute, Kolkata, India; 2 Infectious Diseases and Immunology Division, CSIR-Indian Institute of Chemical Biology, Kolkata, India; University of Birmingham, United Kingdom

## Abstract

In view of the world wide prevalence of *Helicobacter pylori* infection, its potentially serious consequences, and the increasing emergence of antibiotic resistant *H. pylori* strains there is an urgent need for the development of alternative strategies to combat the infection. In this study it has been demonstrated that polyethyleneimine (PEI) functionalized zinc oxide (ZnO) nanoparticles (NPs) inhibit the growth of a metronidazole-resistant strain of *H. pylori* and the molecular basis of the anti-bacterial activity of ZnO-PEI NP has been investigated. The ZnO-PEI NP was synthesized using a wet chemical method with a core size of approximately 3–7 nm. Internalization and distribution of ZnO-PEI NP without agglomeration was observed in *H. pylori* cytosol by electron microscopy. Several lines of evidence including scanning electron microscopy, propidium iodide uptake and ATP assay indicate severe membrane damage in ZnO-PEI NP treated *H. pylori*. Intracellular ROS generation increased rapidly following the treatment of *H. pylori* with ZnO-PEI NP and extensive degradation of 16S and 23S rRNA was observed by quantitative reverse-transcriptase PCR. Finally, considerable synergy between ZnO-PEI NP and antibiotics was observed and it has been demonstrated that the concentration of ZnO-PEI NP (20 µg/ml) that is non-toxic to human cells could be used in combination with sub-inhibitory concentrations of antibiotics for the inhibition of *H. pylori* growth.

## Introduction

*Helicobacter pylori*, a Gram-negative microaerophilic bacterium that colonizes the human gastric mucosa is thought to infect half the world population. In infected individuals, *H. pylori* causes chronic gastric inflammation, which progresses in a significant fraction of cases to peptic ulcer, MALT-lymphoma, or gastric adeno carcinoma [1], the latter being a major cause of cancer-related deaths worldwide. Accordingly, the bacterium was classified as a class I carcinogen by the International Agency for Research on Cancer in 1994. More recently, the results of 12 studies including 1228 cancer cases and 3406 controls showed that *H. pylori*-positive individuals have at least a 6-fold increased risk of developing gastric cancer than *H. pylori*-negative individuals [2]. In this context, the rapid emergence of strains of *H. pylori* resistant to the conventional antibiotic therapy, reported from all over the world, is alarming [3] and emphasizes the urgent need to develop alternative modes of treatment.

Nanoparticles (NPs) have long been known for their antimicrobial properties [4]. Among different inorganic nano-materials, metal and metal oxide NPs are of special interest as they exhibit strong anti-bacterial activity even when administered in very minute doses [5], [6]. The use of ZnO NP is increasing because of its biocompatibility and robustness [7]. ZnO NP with wide band gap (3.3 eV) and high excitonic binding energy (60 MeV) is also considered to be toxicologically safe. Recently U.S. Food and Drug Administration has approved (regulation code: 21CFR182.8991) this material as GRAS (generally recognized as safe). The antimicrobial potential of ZnO NP has been demonstrated against a wide variety of Gram-positive and Gram-negative bacteria; however it has some limitations [8]–[10]. A major limitation of ZnO NP is that it forms aggregates in water due to high surface energy [11] as a consequence of which high concentration of ZnO NP is required for therapeutic purposes. Polymeric agents have been used to improve dispersion of NPs in biological fluids and it has been shown earlier that polyvinyl alcohol (PVA) and chitosan capping over ZnO NP improved its antimicrobial efficacy due to better dispersion of the nanoparticle in aqueous medium [12]–[13].

Polyethyleneimine (PEI) is an aliphatic, cationic branched polymer containing primary, secondary and tertiary amines in its structure. PEI is mostly known for its ability to transfect a wide range of eukaryotic cells and is one of the most efficient agents for non-viral gene delivery applications [14]. In spite of these qualities; the toxicity of PEI towards eukaryotic cells emanating from its cationic nature remained an issue. However, a recent study has demonstrated that the careful selection of the size (10-kDa) of PEI polymer greatly reduces the toxicity [15]–[16]. According to another investigation on FE1 cells, neither PEI-polymers nor nano ZnO crystals could elicit any significant mutagenic activity or oxidative DNA damage in the exposed cells, suggesting their safe use in clinical trials [17]. Apart from these applications PEI can also be used as an efficient membrane permeabilizing agent for Gram-negative bacteria [18]. Although the membrane intervening ability of PEI has been known for quite some time, its antimicrobial effect has been noticed only when it is used as nano formulation [19]. It has been reported that quaternary polyethyleneimine NP has antibacterial action against the cariogenic *Streptococcus mutans* [20]. Also, PEI capping has been demonstrated to improve silver NP colloidal stability and antimicrobial activity [21]. To enhance the antimicrobial activity and stability of ZnO NP in biological fluids we deliberately used PEI capping over ZnO NP and targeted it against *H. pylori*. The rationale behind designing such a nano-conjugate is to enhance the internalization of NP within the bacterial cells as it has been already established that PEI can specifically target lipopolysaccharides (LPS) present in the outer membrane of Gram-negative bacteria [18]. Although the antimicrobial potential of ZnO NP has been investigated against several Gram-negative and Gram-positive bacteria [9]–[10], there is no systematic study on the effect of PEI substituted ZnO NP on any bacterial cell.

The results reported in this study indicate that ZnO-PEI NP has significant toxicity against a metronidazole resistant variant of *H. pylori*. Furthermore, ZnO-PEI NP is relatively stable under a broad range of pH (2 to 7) that *H. pylori* is likely to encounter in the gastric environment, where a pH gradient is thought to exist varying from about pH 2 in the lumen to near neutral pH at the epithelial cell surface [22]. The results also provide some insights into the molecular basis of the anti-bacterial activity of ZnO-PEI NP.

## Materials and Methods

### Ethics Statement

Human blood (10 ml) was collected from volunteers not connected with the study. Recruitment of participants was performed according to protocol approved by the Internal Review Board for the Ethical committee on Human Subjects of the Indian Institute of Chemical Biology, Kolkata to one of the corresponding authors (RC). Written informed consent was obtained from the participants.

### Electron microscopy

The particle size of the prepared nanoparticles were studied using transmission electron microscope (TEM). TEM grids were prepared by placing 10 µL of the diluted and well sonicated sample solutions on a carbon-coated copper grid and dried completely in dust free atmosphere. The bright field electron micrographs of the samples had been recorded on JEM-2010 (device: Orius SC1000) at the accelerating voltage of 200 kV.

### FTIR spectroscopy

FTIR technique was used to determine the binding of PEI to ZnO NP. FTIR scanning was performed in the transmission mode using Perkin-Elmer spectrometer equipped with a DTGS KBr detector and a KBr beam splitter. IR grade KBr was used as scanning matrix. 1–2 mg of fine sample powder and 90–100 mg of KBr powder were mixed and dried completely, then transferred to 13 mm die to make a nearly transparent and homogeneous pallet. All spectra were taken at 4 cm^−1^ resolution, averaged over 20 scans in the range 400 to 4000 cm^−1^.

### Dissolution measurements

Dissolution experiments of ZnO-PEI nanoparticles were performed in a glass beaker at room temperature (∼25°C). In the beaker pH was varied between 2 to 7 and a fixed concentration of ZnO-PEI NP (400 μg/ml) nanoparticles were added with continuous stirring. Aliquots from the supernatant were collected from the beaker, and the solid component was removed by centrifugation. From these samples, the concentration of zinc ions was measured by a Varian 720-ES inductively coupled plasma optical emission spectrometer (ICP-OES). The average of triplicate measures is reported for all dissolution measurements.

### Zeta potential

Surface charge of ZnO and ZnO-PEI NPs were measured using the zeta potential mode of the Malvern Zetasizer Nano ZS. During the experiment pH was varied from 2 to 12, and data were recorded at intervals of single pH.

### Bacterial strains and culture conditions

Metronidazole resistant variant of *Helicobacter pylori* strain 26695 (Minimal inhibitory concentration: >32 μg/ml) were grown on GC agar medium (BD Difco) supplemented with 6.8% defibrinated horse blood (Remel), 8% Horse serum (GIBCO), L-cysteine hydrochloride monohydrate (MP Biomedicals) and isovitalex (BD BBL) at 37°C under micro aerobic conditions (7.5% CO_2_, 5% O_2_) and subcultured every 2 days. For liquid cultures bacteria were harvested from 48 h culture plates and inoculated in Brain Heart Infusion (BHI) medium(BD BBL) supplemented with 10% Horse serum and incubated at 37°C in anaerobic jars under micro aerobic conditions generated by CampyGen bags (Oxoid) with shaking at 120 rpm until the culture reached the desired O.D. Broth cultures of *H. pylori* were treated with different concentrations of ZnO or ZnO-PEI NP, or in combination with different concentrations of antibiotics as mentioned in the text. For all experiments untreated cells were taken as controls.

### Transmission electron microscopy to study ZnO-PEI NP internalization

*H. pylori* cultures (approximately 10^8^ CFU/ml) were incubated with or without ZnO-PEI NP (100 μg/ml) for 3 h. The cells were washed, fixed (2% glutaraldehyde, 2.5% para formaldehyde), treated with 2% OsO_4_ for 2 h and dehydrated using an acetone gradient (30% to 100%). Finally the samples were embedded in resin (CY212 medium grade resin, TAAB laboratories) and blocks were cured at 62°C for 24 h. Ultra thin sections were prepared using a Leica EM UC6 microtome and grids were visualized under a TEM (120kV- Tecnai G2 Spirit Bio Twin).

### Scanning electron microscopic studies

SEM has been used as a tool to analyze the surface topography of the bacteria. *H. pylori* cells were grown to about 10^8^ CFU/ml in BHI medium and then either treated with or without ZnO-PEI NP (100 μg/ml) for 3 h. At regular intervals, samples were removed, fixed with 2% glutaraldehyde, dehydrated with ethanol and examined by SEM (FEI Quanta-200 MK2) with an accelerating voltage of 20 kV. The same instrument is coupled to EDX (energy dispersive X-ray analyzer); the sample preparation for EDX is similar to SEM.

### Propidium iodide uptake assay and ATP assay

*H. pylori* cells were grown to about 10^8^ CFU/ml in BHI medium and then incubated with or without ZnO-PEI NP (100 μg/ml) for 3 h. Bacterial cells were then collected by centrifugation at 4,000 rpm for 5 min and finally re-suspended in Phosphate buffered saline (PBS). The cells were stained with propidium iodide and SYTO9 using the LIVE/DEAD BacLight Bacterial Viability Kit (Invitrogen) following manufacturer's instructions. Stained samples were visualized under a fluorescence microscope. Intracellular ATP levels were measured in NP treated and untreated bacteria using BacTiter-Glo microbial cell viability assay kit (Promega) by measuring luminescence from 10^5^ bacterial cells following manufacturer's protocol. Results obtained are expressed as mean ± standard deviation (SD). The statistical significance of the data was analyzed using the Students *t* test.

### Determination of extracellular ROS by XTT

XTT (2,3-bis-(2-methoxy-4-nitro-5-sulfophenyl)-2H-tetrazolium-5-carboxanilide) (Sigma) assay was used to measure the extracellular (reactive oxygen species) ROS generated by ZnO NP and its analogue. XTT measures radical generation colorimetrically when the generated radicals reduce the tetrazolium dye 2,3-Bis (2-methoxy-4-nitro-5-sulfophenyl)-2H-tetrazolium-5-carboxanilide (XTT) to the highly colored (yellow) XTT formazan. Different concentrations of ZnO NP and its polyethyleneimine capped analogues were incubated in presence 100 μM XTT. After different time intervals the suspension was centrifuged and the supernatant was taken for measuring absorbance at 470 nm.

### Determination of intracellular ROS in bacterial cells

Intracellular ROS was measured using dichlorofluorescein diacetate (H_2_DCF-DA, Sigma). *H. pylori* cells were grown to about 10^8^ CFU/ml in BHI medium and incubated with 30 μg/ml of H_2_DCF-DA for 30 min. H_2_DCF-DA loaded cells were then incubated with or without 100 μg/ml of ZnO-PEI NP for 30 min. The cells were centrifuged (4,000 rpm, 5 min), washed and suspended in PBS. Fluorescence was measured at an excitation wavelength of 485 nm and emission wavelength of 528 nm using a luminescence spectrometer (Model LS55, Perkin Elmer) or cells were visualized under A1R confocal microscope (Nikon). Results obtained were expressed as mean ± SD. The statistical significance of the data was analyzed using the Students *t* test.

### RNA isolation and RNA degradation analysis by qRT-PCR and gel electrophoresis

RNA was isolated from both ZnO-PEI NP (100 μg/ml) treated and untreated *H. pylori* using Trizol reagent (Invitrogen) or SurePrep RNA/DNA/Protein purification kit (Fisher Scientific) following the manufacturers' protocols. Samples were treated with RNase-free DNaseI (1U/μg, Roche) and the amount of RNA in each sample was estimated spectrophotometrically. 1–2 μg of total RNA per lane was electrophoresed on 6% polyacrylamide-7M urea gel. Quantitative real-time RT-PCR (qRT-PCR) was performed using the SYBR Green One-step qRT-PCR kit (Takara) in an iCycler IQ5, Real-time PCR Detection System (Bio-Rad). Equal amount of RNA (40 ng) was used in all experiments. DNase-treated RNA that had not been reverse transcribed was used as a negative control. A dissociation curve was generated at the end of each cycle to verify that a single product was amplified using software provided with the system. Results obtained were expressed as mean ± SD. The statistical significance of the data was analyzed using the Students *t* test.

### MTT assay

Adenogastric sarcoma (AGS) cells were grown in 6 well tissue culture grade plates in RPMI-1640 (GIBCO) media supplemented with 10% (v/v) heat inactivated fetal bovine serum (Pan Biotech) at 37°C in a 5% (v/v) CO_2_ atmosphere. AGS cells upon reaching 70% confluency were treated with ZnO-PEI NP at different concentrations ranging from 0 to 200 μg/ml for 3 h followed by treatment with MTT (3-(4,5-Dimethylthiazol-2-yl)-2,5-diphenyltetrazolium bromide, Sigma) (solubilized in PBS) to a final concentration of 0.5 mg/ml for 2 h. Formazan crystals formed were then solubilized in DMSO and its concentration was measured by a UV-Visible Spectrophotometer (UV-Pharmaspec 1700, Shimadzu) by measuring absorbance at 560 nm. The amount of formazan produced could be directly correlated to the number of viable cells present in each well after treatment. Results were expressed as mean ± SD. The statistical significance of the data was analyzed using the Students *t* test.

### Hemolysis assay

Freshly collected human blood samples stabilized with ethylenediamine tetraacetic acid (EDTA) was centrifuged (1600 rpm, 5 min) and the packed red blood cells (RBC) were washed four times with sterile isotonic PBS. 0.2 ml of packed RBC was diluted to 4 ml with PBS (5% hematocrit), incubated with different concentrations of ZnO-PEI NP in PBS (0.8 ml) at room temperature for 2 h. PBS and distilled water (0.8 ml) were used as negative and positive control respectively. The samples were centrifuged and the absorbance of the supernatant was measured at 541 nm (Cary 4000 UV-Vis spectrophotometer).

### Toxicity of nanoparticles against peripheral blood mononuclear cell (PBMC)

In brief, PBMC were isolated from whole blood by centrifugation through Ficoll-Hypaque solution and were seeded equally in 6 well tissue culture grade plates containing RPMI-1640 media supplemented with 10% FBS (foetal bovine serum, Pan Biotech) and incubated at 37°C in a 5% CO_2_ incubator. PBMC cells were further treated with nano-particles at different concentrations ranging from 5–100 μg/ml for 3 h followed by double staining with fluorescein isothiocyanate (FITC)-conjugated annexin-V and propidium iodide (PI) or 7-aminoactinomycin D (7-AAD) using an apoptosis kit (Invitrogen). % of apoptotic cells were analyzed by flow cytometry using a FACS Calibur (Becton Dickinson) equipped with cell quest pro software (Becton Dickinson). The data were averaged over three identical experiments.

## Results

### Synthesis and characterization of ZnO-PEI NP

The ZnO NPs were prepared according to the modified sol-gel route, subsequently modified with trisodium citrate, followed by PEI capping (Figure S1a, Supporting information) as described previously [23], [24]. The NPs were characterized using high resolution transmission electron microscopy (HRTEM) and Fourier transform infrared spectroscopy (FT-IR). The average size of the NP was found to be 20 nm with a core size ∼7 nm (Figure S1b). The presence of PEI on the surface of ZnO NP was confirmed by FT-IR (Figure S1c), which showed twin peaks at 1587 and 1388 cm^−1^ and broad peak at 3200–3400 cm^−1^ region coming from PEI. The sharp peak at 1587 cm^−1^ is assigned to N-H bending mode of the amine group overlapped with the C-H bending mode of the methylene (-CH_2_) group. The peak at 1388 cm^−1^ is due to the C-H bending vibration of the methyl (-CH_3_) group. The symmetric and asymmetric stretching modes of N-H appear at higher frequency located around 3389 and 3250 cm^−1^. The FT-IR spectrum also has a typical metal oxide (Zn-O) peak at 465 cm^−1^, indicating the formation of ZnO NP.

ZnO NP has a tendency to dissociate at low pH and form Zn^+2^ [11]. To examine the integrity of ZnO-PEI NP, the dissociation of Zn^+2^ from ZnO-PEI NP at different pH was measured by ICP-OES and compared to that from ZnO NP. ZnO-PEI NP has negligible dissociation at pH 7 and remains relatively stable even at pH 2 (only 30% Zn^+2^ leaching was observed, Table S1). In contrast, it has been observed that more than 60% Zn^+2^ leaching occurs in ZnO NP at pH 2.

Zeta potential measures the electro-kinetic potential of the colloid system and magnitude of zeta potential indicates the potential stability of a colloidal system. It is generally considered that the zeta potential value greater than +30 mV or smaller than −30 mV results in a stable solution. Measurement of zeta potential of ZnO NP and ZnO-PEI at different pH suggested that under all pH conditions, ZnO-PEI has higher (positive) zeta potential than ZnO NP (Figure S2). This might be the reason why ZnO-PEI NP dissociates less at lower pH, as zeta potential of NP is directly related to dissolution [11].

### Effect of ZnO-PEI nano particles on *H. pylori*

**Figure 1:**
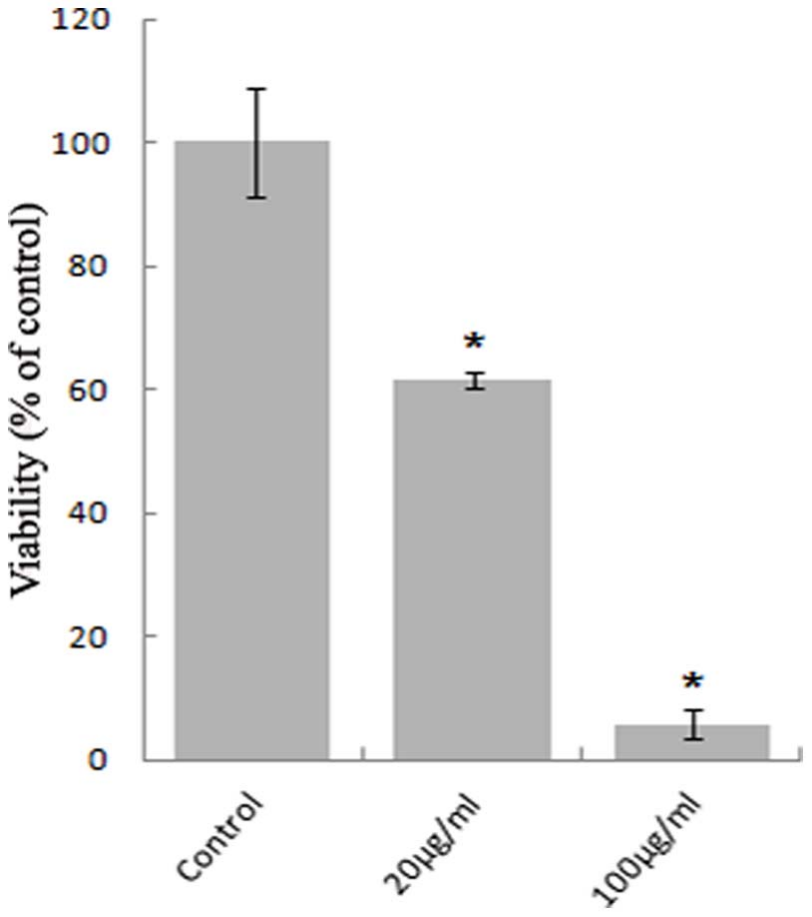
Viability of ZnO-PEI NP treated *H. pylori* strain 26695 grown to the logarithmic phase and treated with different concentrations of ZnO-PEI NP; cells were removed for CFU assay after 24 h of treatment. Results are represented as mean ± SD, * P<0.05.

To examine the antibacterial efficacy of ZnO-PEI NP, *H. pylori* cells were treated with different concentrations of ZnO-PEI NP (0–200 µg/ml). The minimal inhibitory concentration (MIC) was determined to be 100 µg/ml. The effect of the NP on bacterial viability was subsequently examined by CFU assay at regular intervals after treatment of *H. pylori* with different concentrations of ZnO-PEI NP. The results obtained indicated that the maximum growth inhibition (95%) was observed upon treatment of *H. pylori* for 24 h with 100 µg/ml of ZnO-PEI NP (Figure 1). Significant growth inhibition was also observed even after 12 h of treatment (Figure S2). However, at similar concentrations the inhibitory effect of normal ZnO NP on *H. pylori* cells was significantly lower (about 50%) as compared to ZnO-PEI NP (data not shown). The molecular basis of the anti-bacterial activity of ZnO-PEI NP was next investigated.

### Internalization of ZnO-PEI NP into *H. pylori* cells

**Figure 2:**
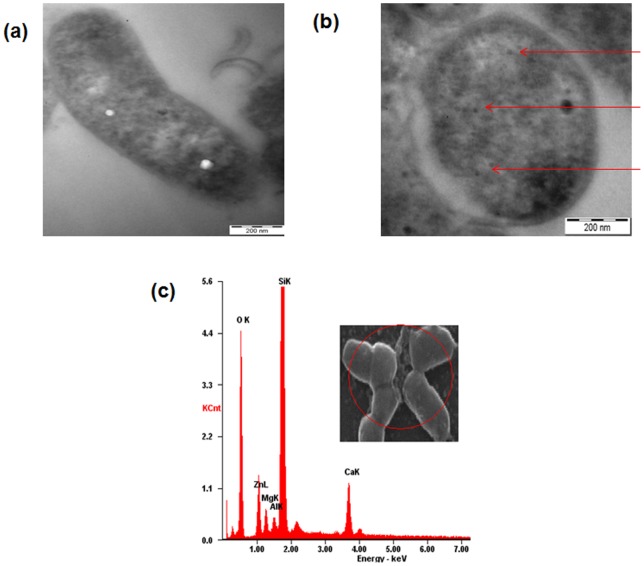
TEM images showing internalization of ZnO-PEI NP into *H. pylori*. (a) Control and (b) NP treated cell, arrow indicates electron dense NPs. (c) EDX spectrum showing characteristic peaks of Zn and O. Inset shows the area where the electron beam was focused.

Internalization of NP into bacterial cells was monitored using transmission electron microscopy (TEM). TEM is a powerful imaging technique, which can distinguish the electron dense ZnO NPs from the background. Control cells show normal cell morphology with no distinct black dots (Figure 2a), whereas ZnO-PEI NP treated cells show signs of internalized NP, numerous dark tiny dots distributed all over the cells could be observed within 3 h of NP treatment (Figure 2b). EDX was also used to monitor the direct attachment of nanoparticles with the bacterial surface [25]. In the spectrum three closely spaced peaks representing Zn, O and Si respectively were observed (Figure 2c). Of these three peaks, the peak corresponding to Si was most intense since the samples were prepared on glass slides. To verify that PEI capping targets the material with greater affinity towards Gram negative bacteria, the adsorption of ZnO-PEI NP on *H. pylori* cells and mammalian cells were compared. It was quite evident that the NP adsorption is much higher in bacteria (Figure S4), and this could be attributed to the presence of lipopolysaccharide (LPS) as mentioned earlier [18].

### ZnO-PEI causes membrane damage of *H. pylori* cells

**Figure 3:**
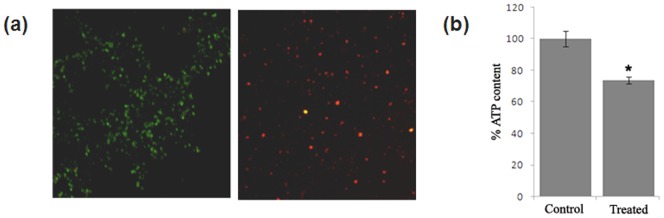
Membrane damage of ZnO-PEI NP treated *H.pylori* strain 26695. Cells were incubated with or without ZnO-PEI NP (100 μg/ml) for 3 h and samples were removed for (a) propidium iodide-SYTO9 staining (untreated cells are shown in the left and treated cells in the right panel) and (b) ATP assay. * P<0.05.

Scanning electron microscopy of *H. pylori* treated with ZnO-PEI NP (100 µg/ml) indicated membrane damage within 3 h of treatment (Figure S5). This observation was further confirmed by a) propidium iodide uptake, and b) estimation of ATP content in ZnO-PEI NP treated and untreated cells. Propidium iodide cannot pass through intact cell membranes, but may freely enter cells with compromised membranes and intercalates into nucleic acids, producing a red fluorescence when excited with light of 488 nm wavelength. On the other hand the green fluorescence dye STYO9 can enter both live and dead cells. When stained with both SYTO9 and propidium iodide, ZnO-PEI NP treated cells mostly generated red fluorescence within 3 h of treatment, indicating severe membrane damage (Figure 3a). The NP-untreated bacterial culture continued to produce only green fluorescence throughout the period examined indicating that the membrane remained intact under the culture conditions used in this study. Intracellular ATP levels in ZnO-PEI NP treated and untreated *H. pylori* cells was next measured, as ATP generation is known to require bacterial inner membrane structural integrity. Approximately 30% depletion in total ATP level was observed in cells treated for 3 h with ZnO-PEI NP (Figure 3b). Taken together these lines of evidence strongly indicated that *H. pylori* undergoes severe membrane damage following treatment with ZnO-PEI NP.

### ZnO-PEI NP imposes oxidative stress on *H. pylori* cells

**Figure 4:**
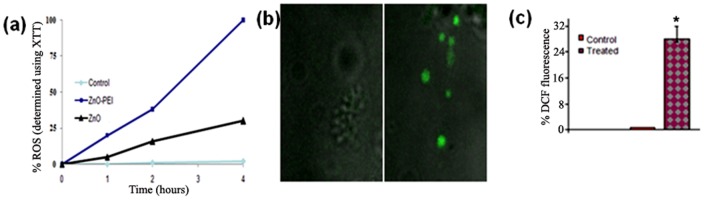
Estimation of ROS. (a) ROS generation by ZnO (from acetate precursor) and ZnO-PEI NPs in aqueous solution was estimated by XTT assay. (b) *H. pylori* strain 26695 preloaded with the fluorescent dye H_2_DCFDA was incubated with or without ZnO-PEI NP (100 μg/ml) for 30 min. (The control cells are shown to the left and the treated cells to the right). (c) (%) Increase in DCF fluorescence on ZnO-PEI treatment measured by fluorescence microscopy. * P<0.05.

In view of the fact that reactive oxygen species (ROS) has been previously proposed to be involved in the genotoxicity and cytotoxicity caused by NPs, ROS generation by ZnO-PEI NP in aqueous suspension was examined by XTT assay. High amounts of ROS were produced by ZnO-PEI NP in aqueous solutions (Figure 4a). The amount of ROS generated was directly proportional to the concentration of ZnO-PEI NP used (Figure S6). Also, at similar concentrations the amount of ROS generated by ZnO NP was much lesser as compared to ZnO-PEI NP (Figure 4a). Next the production of intracellular ROS in *H. pylori* treated with ZnO-PEI NP was examined by H_2_DCF-DA assay. Within 30 min of treatment with 100 µg/ml of ZnO-PEI NP, significantly high amounts of ROS was detected in *H. pylori* cells (Figure 4bc); in a parallel experiment bacteria not treated with NP but subjected to H_2_DCF-DA, did not produce significant fluorescence.

### Morphological transition and degradation of rRNA

**Figure 5:**
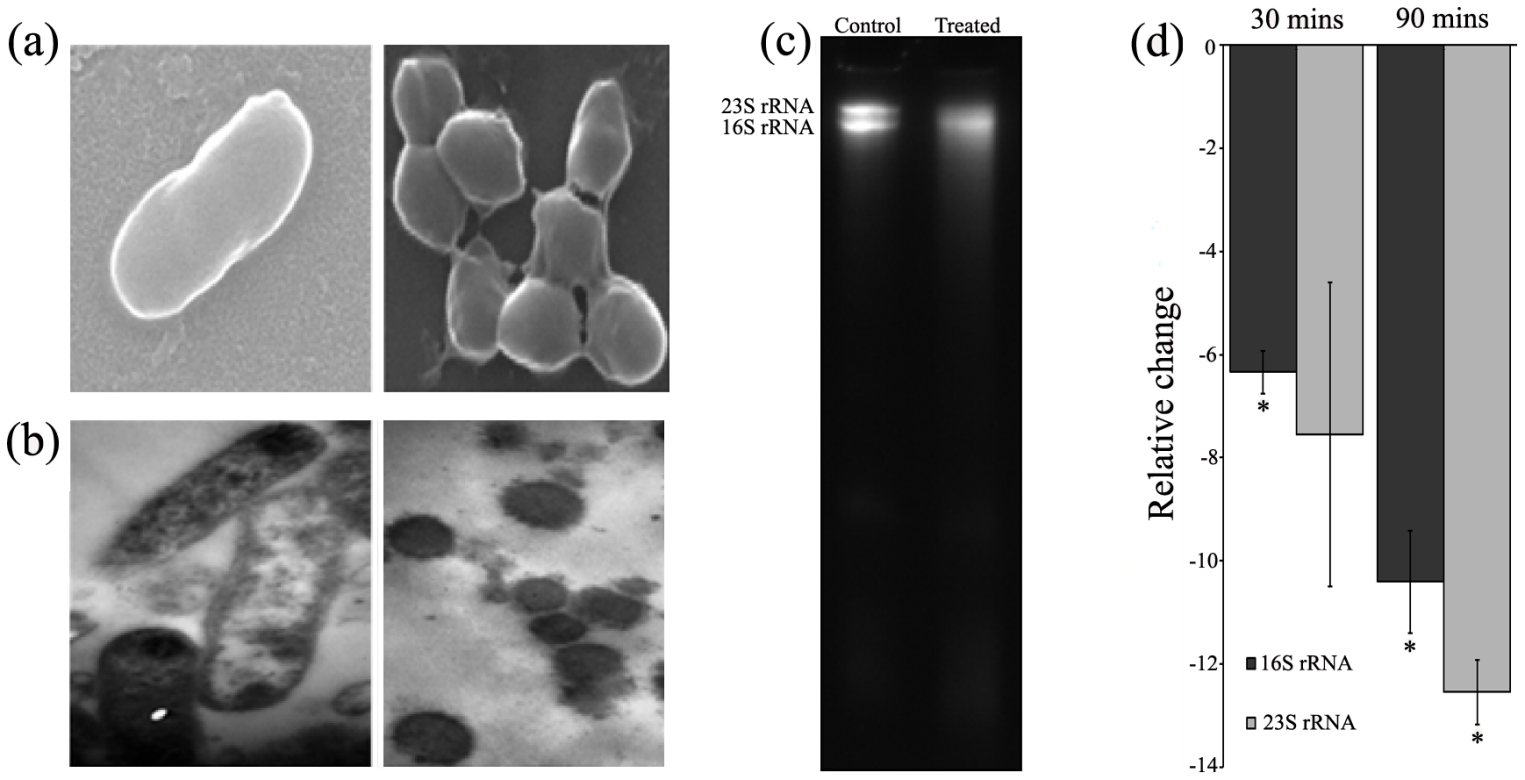
Morphological transition and rRNA degradation. *H. pylori* incubated without or with ZnO-PEI NP for 3 h was visualized by (a) SEM and (b) TEM. (c) RNA was extracted from ZnO-PEI NP treated and untreated *H. pylori* and electrophoresed on 7M urea-6% polyacrylamide gel. In (a), (b) and (c) the left panel shows the control and the right panel shows treated cells. (d) 16S and 23S rRNA was estimated in the RNA samples by qRT-PCR (list of primers are provided in Table S2). * P<0.05.

High levels of intracellular ROS in ZnO-PEI NP treated *H. pylori* was indicative of oxidative stress in the bacteria. A distinctive feature of *H. pylori* is the rapid morphological transition from rod to coccoid on exposure to stress conditions. Indeed, on treatment with ZnO-PEI NP, a time dependent morphological transition was observed in *H. pylori* (Figures 5ab and S7). Furthermore, extensive degradation of 16S and 23S rRNA was observed in the ZnO-PEI NP treated bacteria that paralleled the morphological transition (Figure 5cd), again, the extent of degradation was proportional to the time of treatment with ZnO-PEI NP (Figure 5d). Although it is difficult to establish a causal relationship between rRNA degradation and coccoid conversion it may be relevant that several other conditions like nutrient starvation and other stress conditions lead to concomitant rRNA degradation and conversion to the coccoid form (unpublished observation). Interestingly, when the levels of several stress response genes like sodB (HP0389), catalase (HP0875), fur (HP1027), tsaA (HP1536) and napA (HP0243) were assessed in the ZnO-PEI NP treated bacteria, drastic reduction in their mRNA levels were also observed (Figure S8). Indeed, treatment with different NPs has been shown to result in reduction of stress response gene specific mRNA [26]–[28].

### Toxicity screening

**Figure 6:**
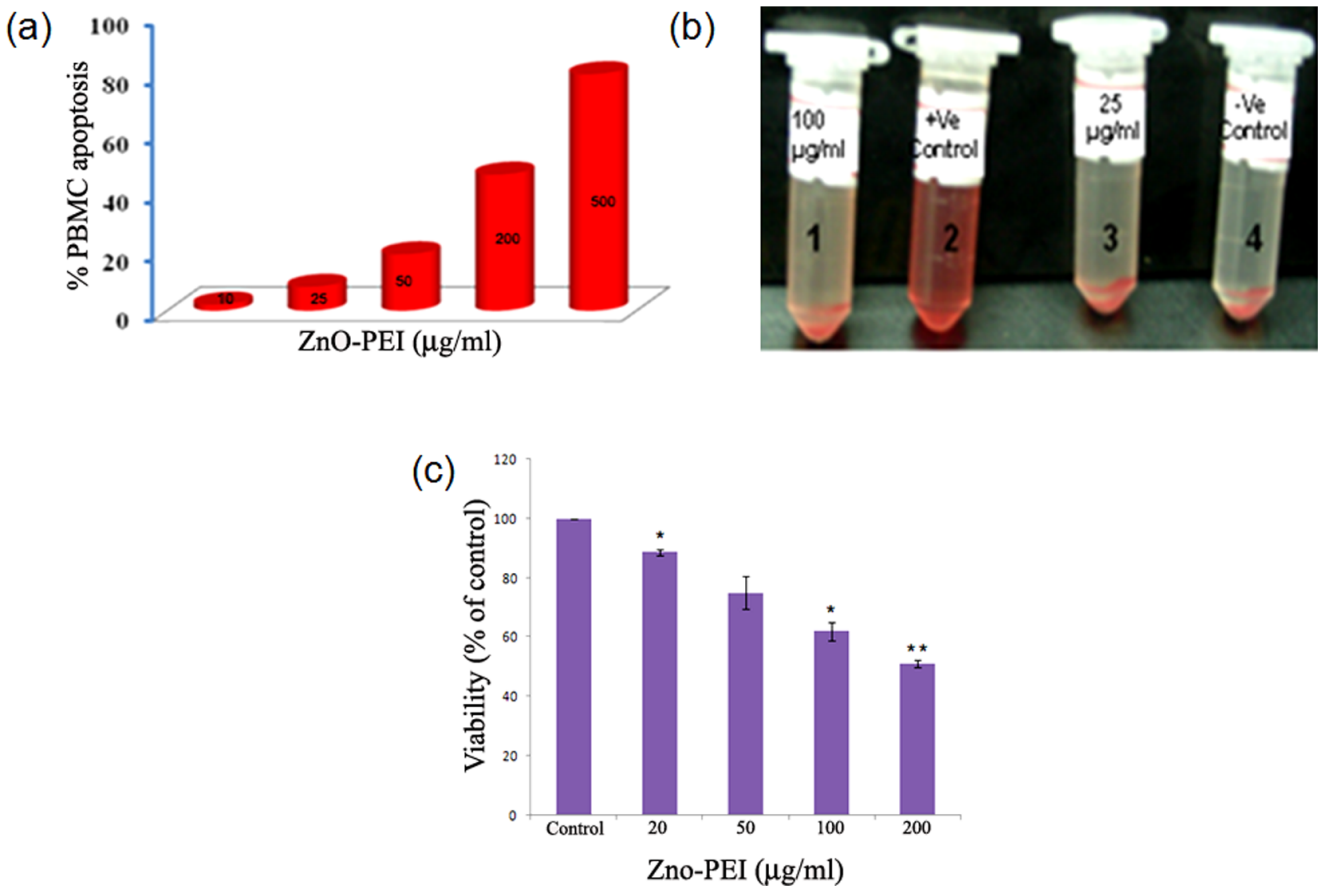
Toxicity of ZnO-PEI NP against different human cells: (a) PBMC, (b) RBC, (c) AGS cells. In (b) tube “2” is the positive control (100% hemolysis); tube “4” is the control, where NP is absent and tubes “3” and “1” represent RBC at different concentrations of NP. * P<0.05, ** P<0.01.

The toxicity of ZnO-PEI NP against gastric adenocarcinoma (AGS) cell line, peripheral blood mononuclear cell (PBMC) and red blood corpuscle (RBC) was next assessed using MTT assay, apoptosis and hemolysis, respectively. In all the three cases a dose dependent cytotoxicity (Figure 6) was observed and AGS cell line was the most sensitive to ZnO-PEI NP. The results indicated that ZnO-PEI NP concentration less than 25 μg/ml may be considered to be toxicologically safe towards the cell lines used in this study.

### Synergistic effect of ZnO PEI NP with antibiotic

**Figure 7:**
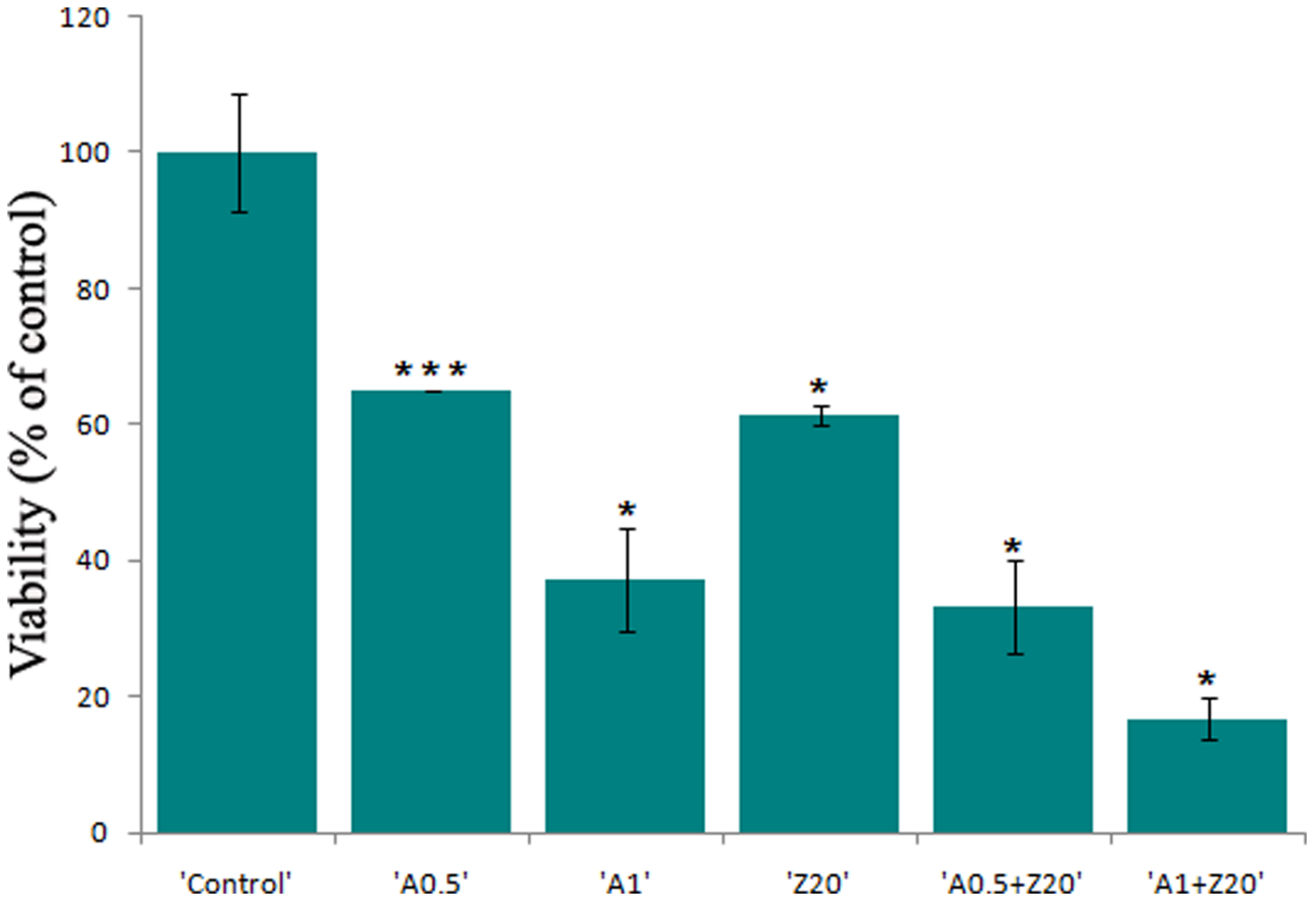
Synergistic effect of ZnO-PEI NP and Ampicillin. *H. pylori* was incubated with ZnO-PEI NP and Ampicillin either alone or in different combinations and O.D. was measured at 600 nm after 24 h of treatment. Ampicilin and ZnO-PEI NP are abbreviated as “A” and “Z”, respectively, followed by a number representing the concentration (µg/ml) used. Error bars represent standard deviation. Results are represented as mean ± SD. * P<0.05, *** P<0.001.

Since the toxicologically safe concentration of ZnO-PEI NP (20 µg/ml) has only moderate anti-bacterial effect (Figure 1), we examined if the effect could be augmented by co-administration with antibiotics. For this purpose, *H. pylori* was incubated with 20 μg/ml ZnO-PEI NP in combination with different concentrations of the antibiotic, ampicillin. While only about 40% growth inhibition of *H. pylori* was observed when incubated with 20 μg/ml ZnO-PEI NP, more than 80% inhibition was observed when the ZnO-PEI NP was used in combination with 1 μg/ml of ampicillin (Figure 7). It may be noted that the minimum inhibitory concentration (MIC) of ampicillin was more than 5 μg/ml. These results indicated notable synergy between ZnO-PEI NP and antibiotics in killing *H. pylori*.

## Discussion

In this study we have demonstrated that ZnO-PEI NP is efficiently internalized into *H. pylori* cells, where it generates considerable amounts of intracellular ROS resulting in membrane damage, degradation of stable RNA, morphological transition to the coccoid form and finally loss of viability. The antimicrobial efficacy of ZnO NP has been well known over a decade [9], however, PEI substituted ZnO nanoparticles used in this study have several advantages over the ZnO NP. Firstly, it disperses better in aqueous media than ZnO NP. Secondly, the release of Zn^+2^ ion, which is toxic to animal cells [29], is lower from ZnO-PEI NP than from ZnO NP, especially at low pH characteristic of the stomach. Also, as suggested by zeta potential measurements (Figure S2), at low pH the stability of ZnO-PEI NP is higher than that of ZnO NP.

The use of zinc compounds for the treatment of *H. pylori* is known [30]; in fact the protective effect of Zinc monoglycerolate (a slow releasing Zn^2+^ complex) on the gastrointestinal mucosa of rodents is well established [31]. Release of Zn^+2^ ions from ZnO NP has been postulated to be largely responsible for the antimicrobial activity of ZnO NP [32]. However, since ZnO-PEI NP has much higher anti-*H. pylori* activity but releases much lower amounts of Zn^+2^ ions as compared to ZnO NP (Table S1), it is attractive to hypothesize that the anti *H. pylori* activity of ZnO-PEI NP may not be solely due to release of Zn^+2^ ions. Also, direct interaction of NPs with bacterial cell surfaces and subsequent membrane damage is considered to contribute to antimicrobial activity of NPs [33]. The direct evidence of ZnO-PEI NP association with the bacterial membrane came from EDX spectroscopy (Figure 1c) and the resulting severe membrane damage has been demonstrated by microscopy as well as propidium iodide uptake assays (Figure 3a). When we analyzed the bacterial membrane after 3 h of nanoparticle treatment we found severe damage in membrane topography along with irregular surface formation (Figures S4). It was also interesting to find that ZnO-PEI NP generates much higher amount of ROS compared to ZnO NP (Figure 4a). ROS is a well known factor influencing NP mediated bacterial killing [34] as ROS can effectively damage bacterial membranes [10] and it also has the ability to degrade nucleic acids. These phenotypes have been demonstrated in ZnO-PEI NP treated *H. pylori* suggesting that probably ROS generation is the principal cause of the anti-*H. pylori* effect of ZnO-PEI NP. In *H. pylori*, diverse stress conditions are known to cause morphological transition to the coccoid form. Likewise, the intracellular ROS generated upon treatment of *H. pylori* with ZnO-PEI NP would impose severe stress on the bacteria, hence, as expected *H. pylori* undergoes conversion to the coccoid form under these conditions. It has been recently demonstrated that *Campylobacter jejuni*, which is closely related to *H. pylori*, also undergoes conversion to the coccoid form upon treatment with ZnO NP [35].Toxicity of NP always remains a controversial issue and the toxic concentration of NPs varies depending on the nature of the cell lines. Recently Akhtar *et al*. have shown that ZnO NPs have distinct effects on mammalian cell viability, killing cancer cells (HepG2, A549 and BEAS-2B), while posing no effect on normal cells (rat astrocytes and hepatocytes) [36]. Also, more ROS is generated in the presence of NP in fast proliferating cells as compared to their slowly proliferating counterparts; so they are more prone to oxidative damage. Similar results were obtained in this study, among the three cell lines studied, AGS has the maximum proliferation potential, followed by PBMC and RBC. ZnO-PEI NP toxicity was maximum towards AGS, followed by PBMC and RBC (Figure 6). Fortunately we found that ZnO-PEI NP concentration less than 25 μg/ml does not show significant toxicity against AGS cells. At this concentration, in combination with sub-lethal concentrations of antibiotics, ZnO-PEI NP produces very significant inhibition of *H. pylori* (Figure 7). It is likely that the synergistic effect of ZnO-PEI NP and antibiotics is due to membrane damage produced by the NP that allows better access of the antibiotics to their intracellular targets.

**Figure 8:**
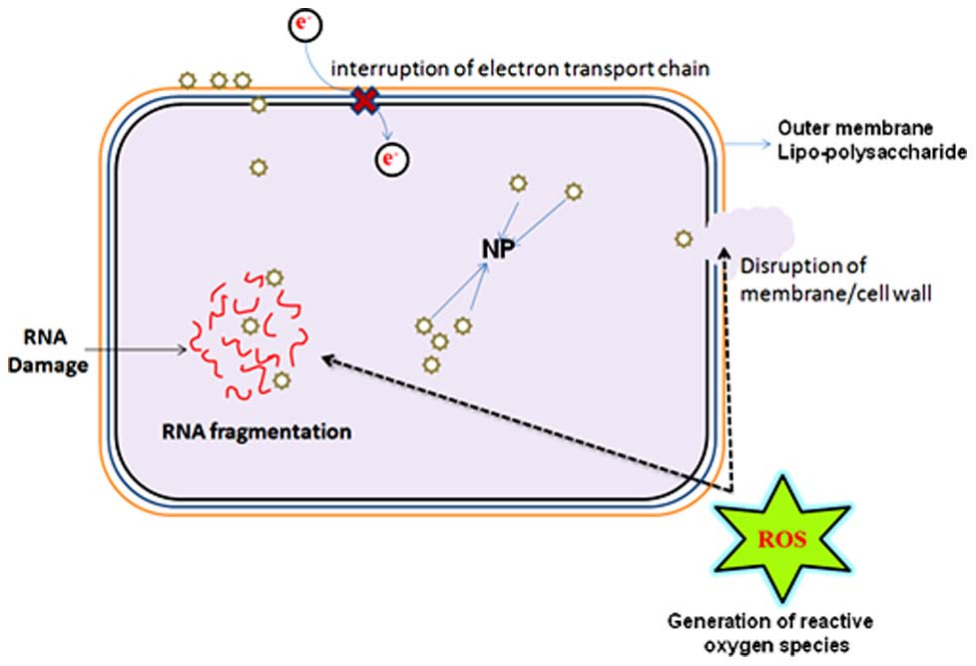
Schematic representation of the mechanism of antimicrobial activity of ZnO-PEI NP.

In this report, we comprehensively show how ZnO-PEI NP elicits its antimicrobial action against one of the deadliest pathogens of stomach, *H. pylori* (Figure 8). This study has several salient features, which are summarized as follows. i) ZnO NP has the intrinsic ability to arrest *H. pylori* growth, and PEI capping significantly accelerates this antibacterial potential. ii) ZnO-PEI NP mainly damages bacterial cells through generation of ROS; however, direct interaction between NP and the bacterial cell is also visible. iii) ROS mediated stress (oxidative stress) causes a morphological transformation in the bacterium (rod to coccoid transition), and also causes the 16S rRNA and 23S rRNA levels to diminish rapidly. iv) NP in combination with antibiotic (ampicillin) shows enhancement in antibacterial activity. Altogether these results indicate a toxicologically safe application of ZnO-PEI NP against a drug resistant *H. pylori* species. Nanotechnology is bestowed with the immense promise for the manipulation of the structure and chemistry of surfaces to inhibit bacterial colonization and we strongly believe that this investigation will pave the way for new directions towards the synthesis of new generation antimicrobials.

## Supporting Information

Figure S1 **Synthesis and characterization of ZnO-PEI NP.** (a) Schematic representation of ZnO-PEI NP synthesis. (b) HR-TEM images of ZnO-PEI NP. (c) FT-IR spectrum of ZnO-PEI nanoparticle in the transmittance mode [24].
(TIF)

Figure S2 **Zeta potential values for ZnO and ZnO-PEI at different pH.**
(TIF)

Figure S3 **Degree of ZnO-PEI NP adsorption on** ***Helicobacter pylori*** **cells compared to PBMC cell (as determined using EDX).** In both the cases the same amount of ZnO-PEI NP was used.
(TIF)

Figure S4 **Effect of ZnO-PEI NP on the growth of** ***H. pylori*** **cells at different time points.**
(TIF)

Figure S5 **SEM image indicating membrane damage in** ***H. pylori*** **on treatment with ZnO-PEI NP (100** **μg/ml) for 3** **h.** Arrow indicates the site of membrane damage.
(TIF)

Figure S6 **Concentration dependent increase of ROS by ZnO-PEI NP as estimated by XTT assay.**
(TIF)

Figure S7 **Morphological transition (rod to coccid) of** ***H. pylori*** **cells in presence of ZnO-PEI NP (100** **μg/ml) with increasing time intervals.**
(TIF)

Figure S8 **Relative change in the mRNA levels of several stress response genes in** ***H. pylori*** **cells treated with 100** **μg/ml of ZnO-PEI NP for 90** **min as determined by qRT-PCR.** * P<0.05, *** P<0.001.
(TIF)

Table S1 **The extent of Zn^+2^ release (μg/ml) by ZnO-PEI and ZnO NP at different pH values.**
(DOC)

Table S2 **List of primer sequences used for qRT-PCR.**
(DOC)
