# Supplementary figures and images for: The Molecular Basis of Inactivation of Metronidazole-Resistant Helicobacter pylori Using Polyethyleneimine Functionalized Zinc Oxide Nanoparticles
Source: PLoS One. 2013 Aug 8;8(8):e70776. doi: 10.1371/journal.pone.0070776 (PMC3738536; doi:10.1371/journal.pone.0070776)

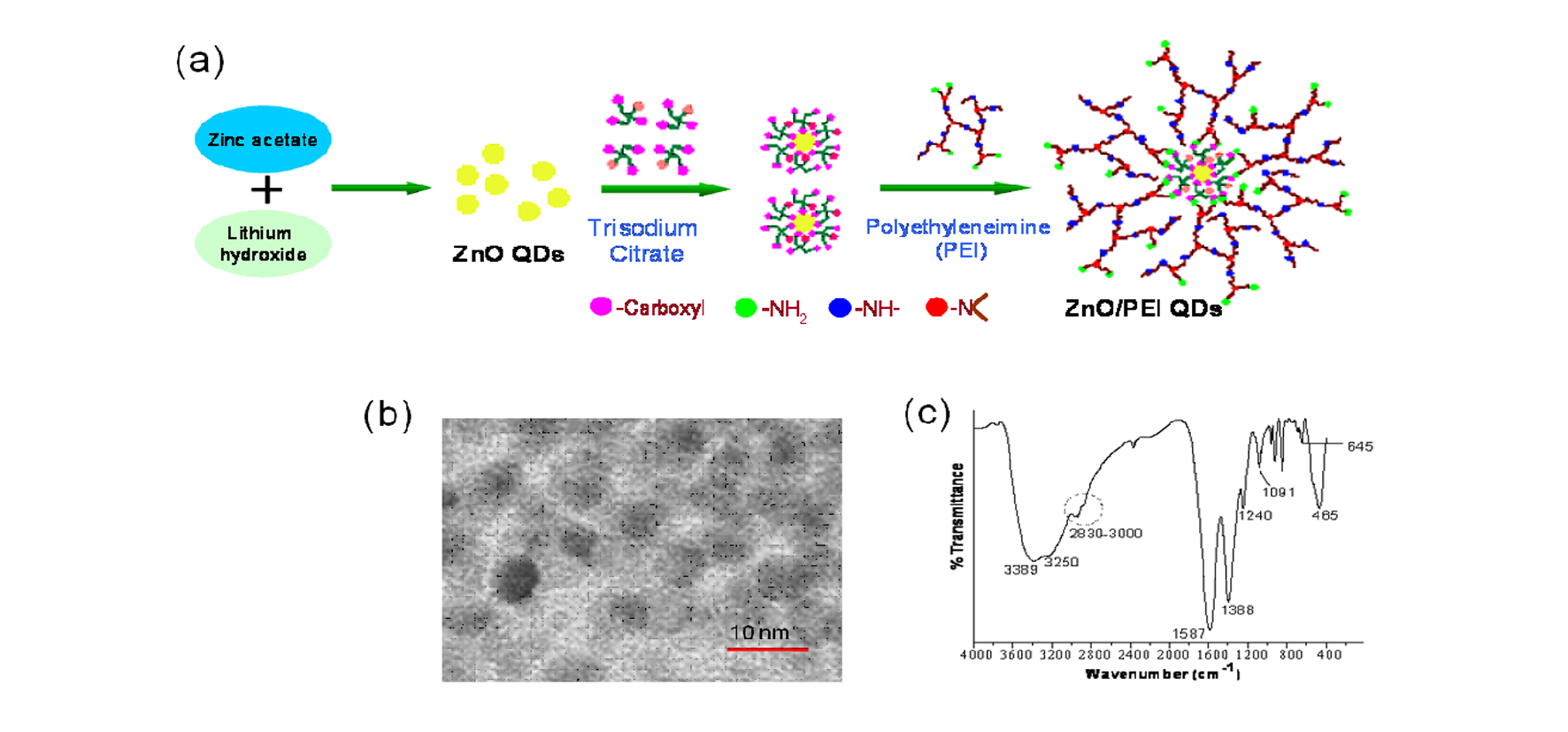

Supplement: Figure S1 — Synthesis and characterization of ZnO-PEI NP. (a) Schematic representation of ZnO-PEI NP synthesis. (b) HR-TEM images of ZnO-PEI NP. (c) FT-IR spectrum of ZnO-PEI nanoparticle in the transmittance mode [24]. (TIF) [file pone.0070776.s001.tif]

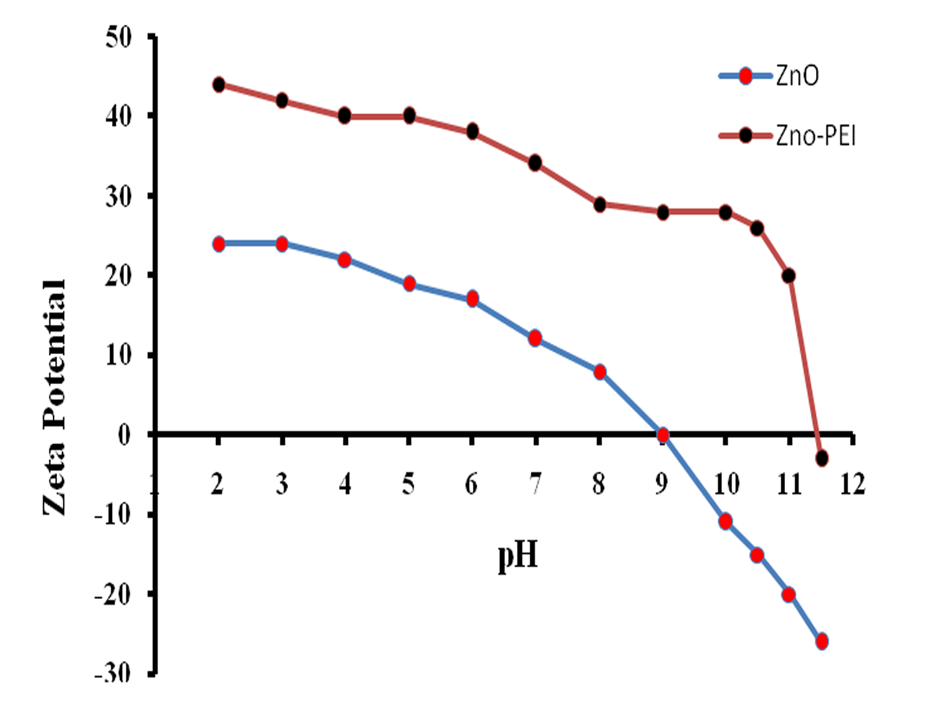

Supplement: Figure S2 — Zeta potential values for ZnO and ZnO-PEI at different pH. (TIF) [file pone.0070776.s002.tif]

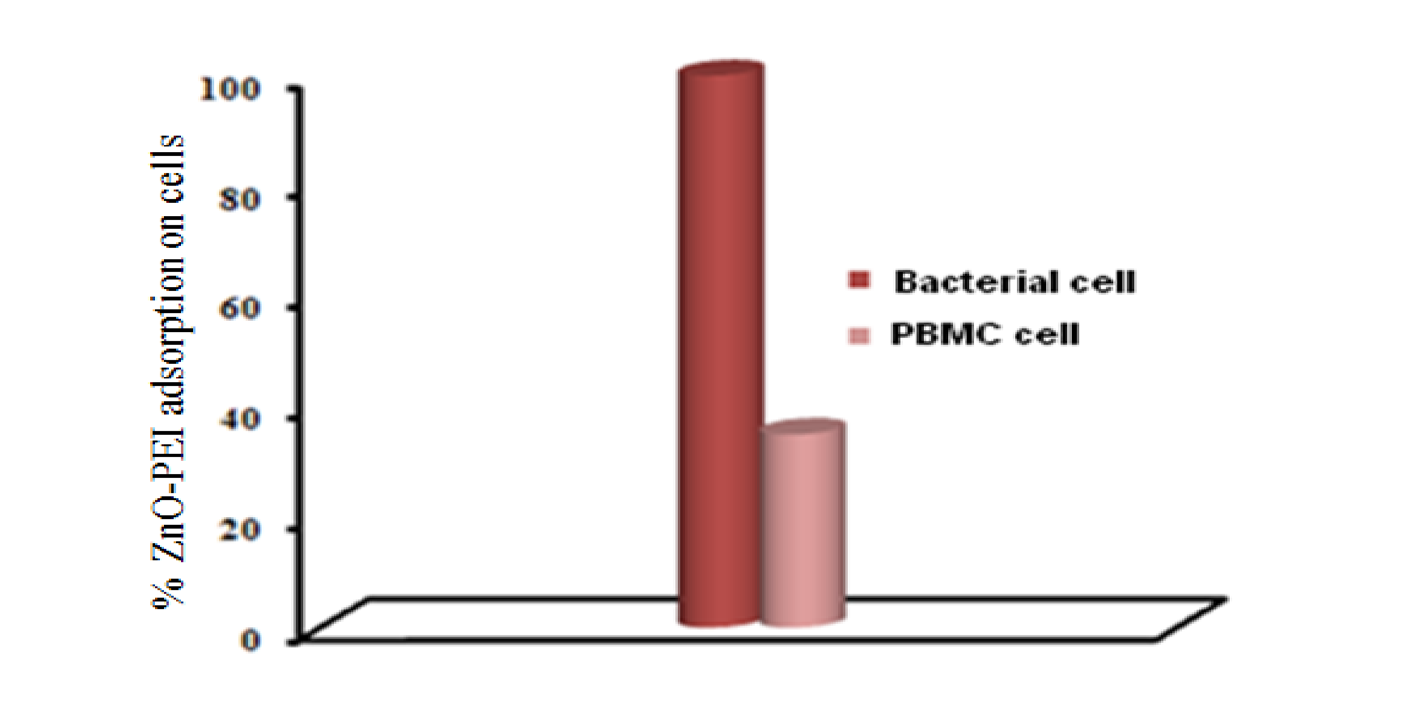

Supplement: Figure S3 — Degree of ZnO-PEI NP adsorption on Helicobacter pylori cells compared to PBMC cell (as determined using EDX). In both the cases the same amount of ZnO-PEI NP was used. (TIF) [file pone.0070776.s003.tif]

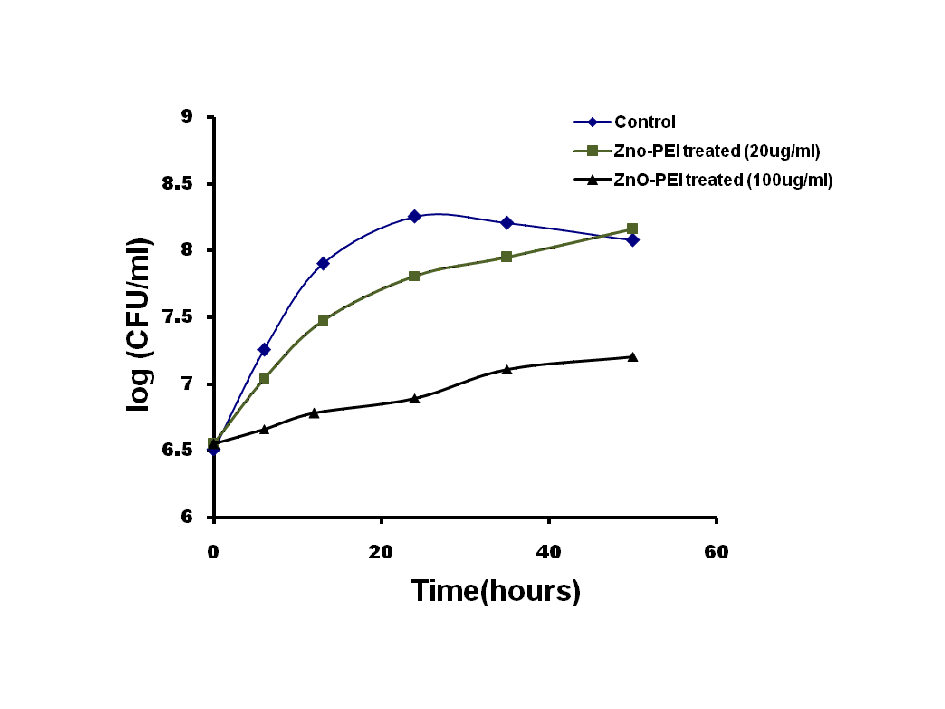

Supplement: Figure S4 — Effect of ZnO-PEI NP on the growth of H. pylori cells at different time points. (TIF) [file pone.0070776.s004.tif]

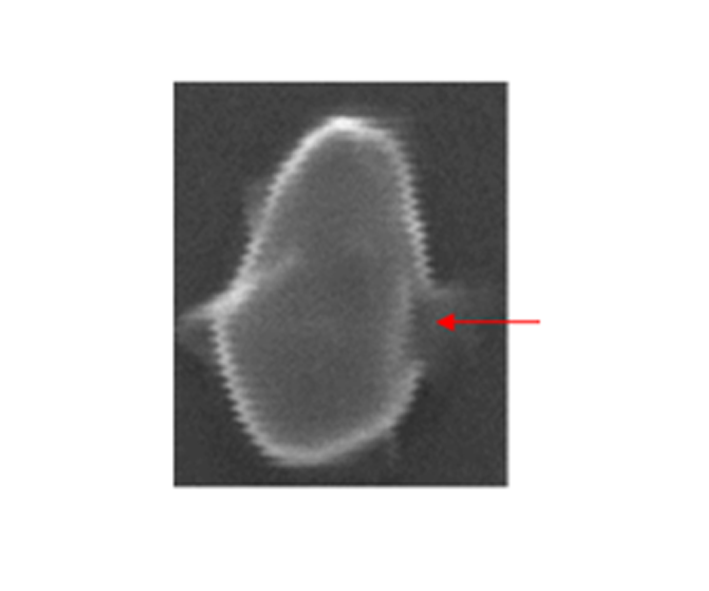

Supplement: Figure S5 — SEM image indicating membrane damage in H. pylori on treatment with ZnO-PEI NP (100 μg/ml) for 3 h. Arrow indicates the site of membrane damage. (TIF) [file pone.0070776.s005.tif]

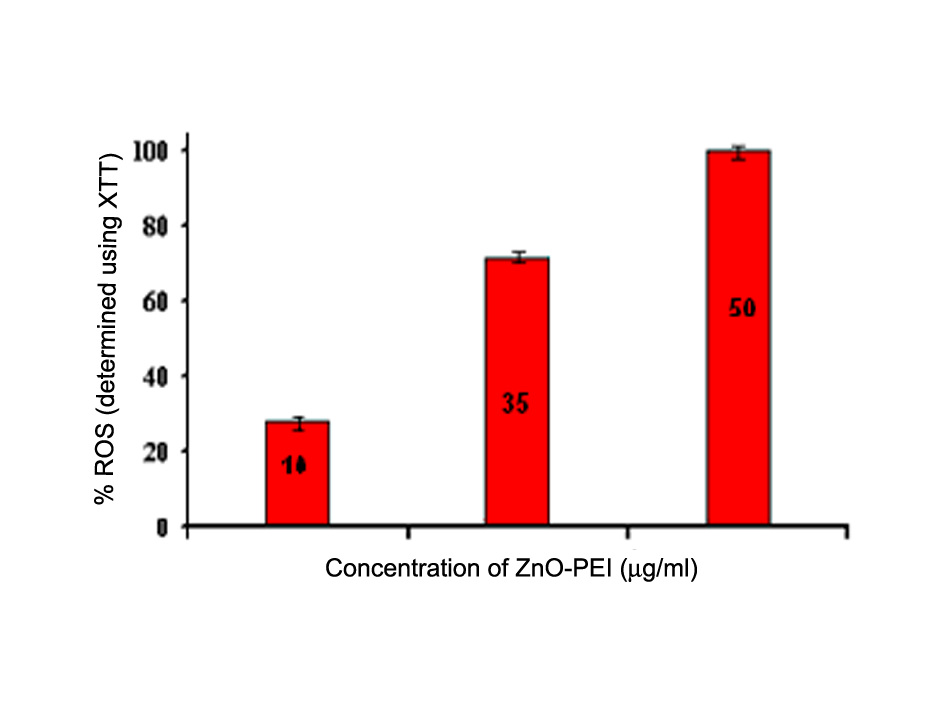

Supplement: Figure S6 — Concentration dependent increase of ROS by ZnO-PEI NP as estimated by XTT assay. (TIF) [file pone.0070776.s006.tif]

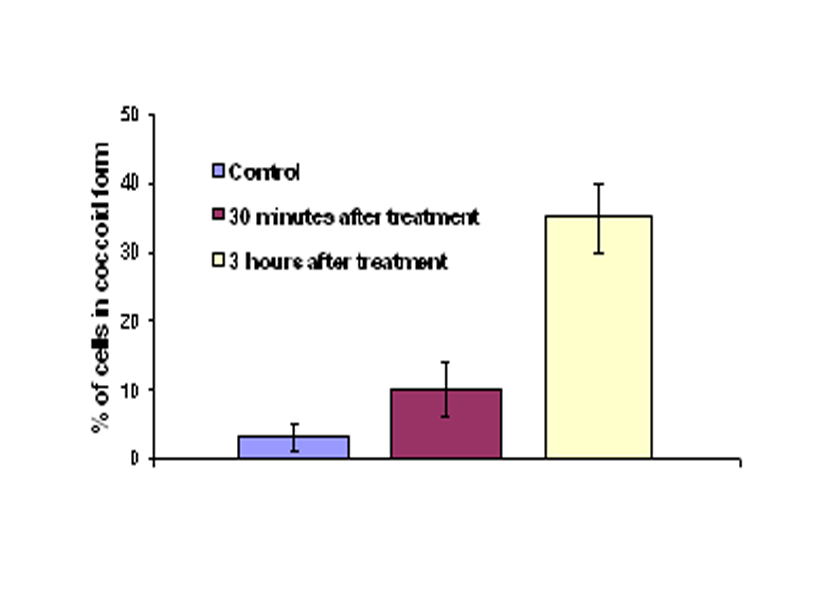

Supplement: Figure S7 — Morphological transition (rod to coccid) of H. pylori cells in presence of ZnO-PEI NP (100 μg/ml) with increasing time intervals. (TIF) [file pone.0070776.s007.tif]

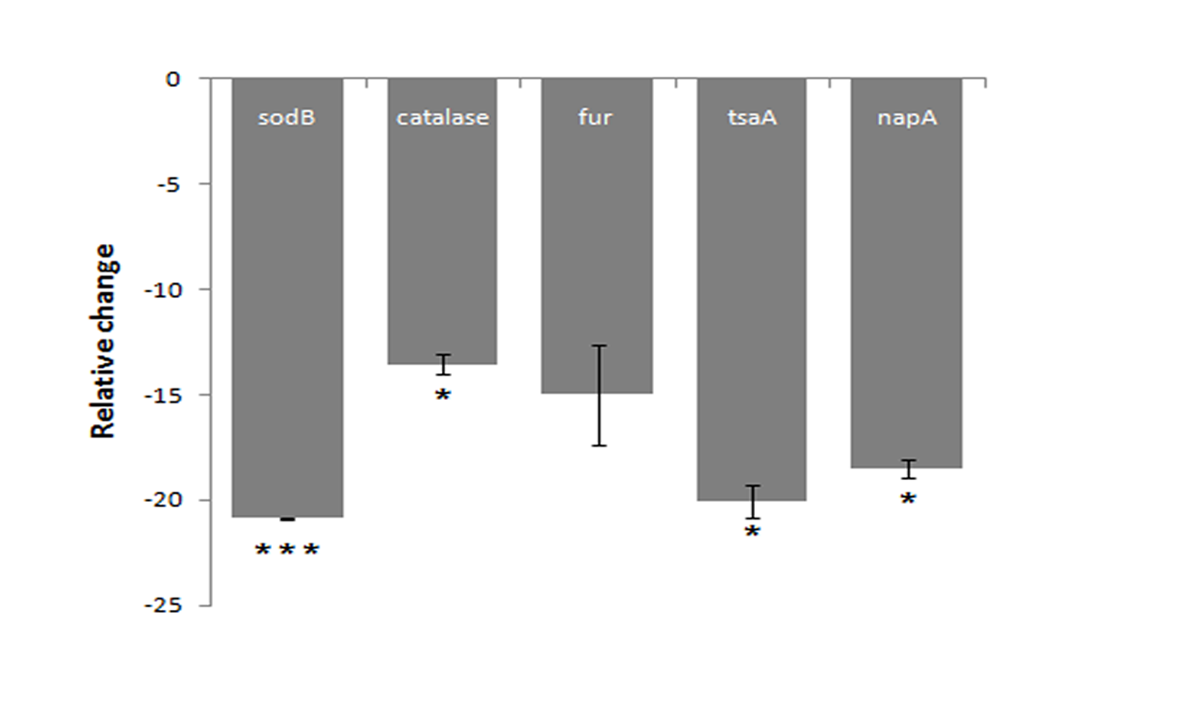

Supplement: Figure S8 — Relative change in the mRNA levels of several stress response genes in H. pylori cells treated with 100 μg/ml of ZnO-PEI NP for 90 min as determined by qRT-PCR. * P<0.05, *** P<0.001. (TIF) [file pone.0070776.s008.tif]
