# Supplementary material for: The Molecular Basis of Inactivation of Metronidazole-Resistant Helicobacter pylori Using Polyethyleneimine Functionalized Zinc Oxide Nanoparticles
Source: PLoS One. 2013 Aug 8;8(8):e70776. doi: 10.1371/journal.pone.0070776 (PMC3738536; doi:10.1371/journal.pone.0070776)
Supplement: Table S1 — The extent of Zn+2 release (μg/ml) by ZnO-PEI and ZnO NP at different pH values. (DOC) [file pone.0070776.s009.doc]

|  | **Amount of Zn^2+^ released (μg/ml) (% release) at pH** | | | |
| --- | --- | --- | --- | --- |
|  | **2** | **3** | **5** | **7** |
| **ZnO-PEI** | **120 (30%)** | **100 (25%)** | **15 (4%)** | **6.5 (1.6%)** |
| **ZnO** | **64%** | **(45%)^11^** | **(25%)^11^** | **22.6 (6.5%)** |

**Measured by inductively coupled plasma optical emission spectroscopy (ICP-OES). Sample concentrations were 400 μg/ml.**

**Table S1. The extent of Zn^2+^ released (μg/ml) by ZnO-PEI and ZnO NP at different pH values.**
