# Supplementary material for: The Molecular Basis of Inactivation of Metronidazole-Resistant Helicobacter pylori Using Polyethyleneimine Functionalized Zinc Oxide Nanoparticles
Source: PLoS One. 2013 Aug 8;8(8):e70776. doi: 10.1371/journal.pone.0070776 (PMC3738536; doi:10.1371/journal.pone.0070776)
Supplement: Table S2 — List of primer sequences used for qRT-PCR. (DOC) [file pone.0070776.s010.doc]

| Primer | Sequence (5'-3') |
| --- | --- |
| 16S rRNA Forward primer | GGGATAGTCAGTCAGGTGTG |
| 16S rRNA Reverse primer | ACTAGCATCCATCGTTTAGG |
| 23S rRNA Forward primer | CATGCGCTGAAAATATAACG |
| 23S rRNA Reverse primer | CCGACTAACCCTACGATGAC |
| sodB Forward primer | CCACGATTTTTATTGGGATTG |
| sodB Reverse primer | TAACTGGGGTTTGAGCGTTG |
| catalase Forward primer | AGAGGTTTTGCGATGAAG |
| catalase Reverse primer | CGTTTTTGAGTGTGGATG |
| fur Forward primer | GAAGTGGTGAGCGTTTTGTATC |
| fur Reverse primer | TTCATTCTGGCGGTTTTCA |
| tsaA Forward primer | CGGTGCGATTCTTTTCTTCT |
| tsaA Reverse primer | GCCTTTTTCTACAGGGGTGTT |
| napA Forward primer | TGTGAAAGGCACCGATTTTT |
| napA Reverse primer | CGAGTGAGTTTGATCGCTTC |

**Table S2. List of primer sequences used for qRT-PCR**
